# Novel predictive epigenetic signature for temozolomide in non-G-CIMP glioblastomas

**DOI:** 10.1186/s13148-019-0670-9

**Published:** 2019-05-14

**Authors:** An-An Yin, Ya-Long He, Amandine Etcheverry, Yu-He Liu, Marc Aubry, Jill Barnholtz-Sloan, Bo-Lin Liu, Jean Mosser, Zi-Fan Lu, Xiang Zhang

**Affiliations:** 1 Department of Neurosurgery, Xijing Institute of Clinical Neuroscience, Xijing Hospital, Air Force Medical University, West Road, No. 169 Xi’an, Changle, 710032 Shaanxi Province China; 2 State Key Laboratory of Cancer Biology, Department of Pharmacogenomics, School of Pharmacy, Air Force Medical University, Xi’an, Shaanxi Province China; 3 Department of Neurosurgery, the 960th Hospital of the People’s Liberation Army, Taian, Shandong Province China; 4 CNRS, UMR 6290, Institut de Génétique et Développement de Rennes (IGdR), 35043 Rennes, France; 5 UEB, UMS 3480 Biosit, Faculté de Médecine, Université Rennes 1, 35043 Rennes, France; 6 CHU Rennes, Service de Génétique Moléculaire et Génomique, 35033 Rennes, France; 7 Case Comprehensive Cancer Center, Case Western Reserve University, Cleveland, OH USA; 8 Department of Neurosurgery, Tangdu Hospital, Air Force Military Medical University, Xi’an, Shaanxi Province China; 9 Plate-forme Génomique Santé Biosit, Université Rennes 1, 35043 Rennes, France

**Keywords:** Glioma-CpGs island methylator phenotype, Glioblastoma, Temozolomide, Predictive biomarker, DNA methylation

## Abstract

**Objective:**

To identify novel epigenetic signatures that could provide predictive information that is complementary to promoter methylation status of the O-6-methylguanine-DNA methyltransferase (MGMT) gene for predicting temozolomide (TMZ) response, among glioblastomas (GBMs) without glioma-CpGs island methylator phenotype (G-CIMP)

**Methods:**

Different cohorts of primary non-G-CIMP GBMs with genome-wide DNA methylation microarray data were included for discovery and validation of a multimarker signature, combined using a RISK score model. Different statistical analyses and functional experiments were performed for clinical and biological validation.

**Results:**

By employing discovery cohorts with radiotherapy (RT) and TMZ versus RT alone and a strict multistep selection strategy, we identified seven CpGs, each of which was significantly correlated with overall survival (OS) of non-G-CIMP GBMs with RT/TMZ, independent of age, MGMT promoter methylation status, and other identified CpGs. A RISK score signature of the 7 CpGs was developed and validated to distinguish non-G-CIMP GBMs with differential survival outcomes to RT/TMZ, but not to RT alone. The interaction analyses also showed differential outcomes to RT/TMZ versus RT alone within the RISK score-based subgroups. The signature could also improve the risk classification by age and MGMT promoter methylation status. Functional experiments showed that HSBP2 appeared to be epigenetically regulated by one identified CpG and was associated with TMZ resistance, but it was not associated with cell proliferation or apoptosis in GBM cell lines. The predictive value of the single CpG methylation of HSBP2 by pyrosequencing was observed in a local cohort of isocitrate dehydrogenase 1 (IDH1) ^R132H^ wild-type GBMs.

**Conclusions:**

This novel epigenetic signature might be a promising predictive (but not a general prognostic) biomarker and be helpful for refining the MGMT-based guiding approach to TMZ usage in non-G-CIMP GBMs.

**Electronic supplementary material:**

The online version of this article (10.1186/s13148-019-0670-9) contains supplementary material, which is available to authorized users.

## Introduction

Glioblastomas ᅟ (GBMs) are a group of clinically refractory disease with apparent intertumor heterogeneity and a generally poor prognosis [1]. Over the past decade, despite extensive explorations on novel therapeutic strategies such as anti-angiogenic therapy [2, 3], immunotherapy [4], and the use of tumor treating fields (TTFs) [5], the combination of radiotherapy (RT) and temozolomide (TMZ) had remained the standard adjuvant treatment for newly diagnosed GBMs [6]. Unfortunately, these tumors often have variable responses to TMZ, and some do not benefit from the combined RT/TMZ treatment. The promoter methylation status of the O-6-methylguanine-DNA methyltransferase (MGMT), encoding a DNA repair enzyme that confers resistance to alkylating drugs, has been by far the most informative predictive biomarker for TMZ outcome in GBMs [7]. However this single-gene methylation status has limitations for clinical utility, especially for guiding the choice of TMZ in unmethylated tumors [8]. Therefore, novel predictive biomarkers with a high predictive value that are independent of MGMT promoter methylation status could be useful. In this study, we investigated the major subgroup of GBMs that do not have the glioma-CpGs island methylator phenotype (G-CIMP), which is exclusively featured by the absence of isocitrate dehydrogenase (IDH) mutations (mostly IDH1^R132H^) [9]. We developed a novel 7-CpG signature using genome-wide DNA methylation data; the signature may confer predictive information for TMZ usage that is complementary to MGMT promoter methylation status. In addition, we selected the HSBP2 gene for further analysis, the expression of which might be epigenetically regulated by one of the 7 CpGs. Functional studies on this epigenetically regulated gene (HSBP2) provide additional biological and clinical insights to the multimarker signature.

## Methods

### Molecular datasets from Rennes and Angers University Hospitals

A total of 125 primary non-G-CIMP GBMs were collected between 2004 and 2013 from the Neurosurgery Departments of Rennes and Angers University Hospitals (RAUH), including a new cohort of 77 samples (*RAUH-new cohort*) and a published cohort of 48 samples (*RAUH-GSE22891*) [10]. Snap-frozen samples were collected at the time of surgery, following written informed consent, in accordance with the French regulations and the Helsinki Declaration. Initial histological diagnoses were confirmed by a central review panel including at least two neuropathologists. Degree of surgical resection was defined by MRI 72 h after surgery. All patients were treated with RT plus concurrent and adjuvant TMZ. Only samples with > 80% tumor cells were selected for microarray profiling and molecular detection. DNA and RNA were isolated as previously described [10]. DNA methylation and gene expression microarrays were performed according to the manufacturer’s instructions. Specifically, *RAUH*-*new cohort* was profiled by the Infinium HumanMethylation450k BeadChip for DNA methylation (deposited in The ArrayExpress at http://www.ebi.ac.uk/arrayexpress/ under the accession number of E-MTAB-4969). Image processing and intensity data extraction were performed within Genome Studio (Illumina Inc.). The novel BMIQ (Beta MIxture Quantile dilation) algorithm was used for intra-array normalization [11]. The methylation data of each CpG is summarized as *β* value, ranging from 0 (completely unmethylated) to 1 (completely methylated). All but two samples were also profiled by Agilent Whole HumanGenome 8 × 60 K Microarray Kit (Agilent Technologies) for gene expression. Expression intensity was log2 transformed and normalized (scale 50th percentile and baseline transformation) within GeneSpring GX software (Agilent Technologies). DNA methylation and gene expression profiling for *RAUH-GSE22891* were reported previously (deposited in Gene Expression Omnibus [GEO] at https://www.ncbi.nlm.nih.gov/geo/ under the accession number of GSE22891) [10]. G-CIMP status was determined by *k*-means (*k* = 3) clustering on the 1503 featured probes reported by The Cancer Genome Atlas (TCGA) [9]. MGMT promoter methylation status was determined using a logistic regression model based on two Illumina array probes, i.e., cg12434587 and cg12981137 [12].

### Molecular datasets from public databases

Additional DNA methylation or gene expression data of primary non-G-CIMP GBMs were obtained from public databases, including the clinically annotated cohort from TCGA (*TCGA-Brennan et al-RT/TMZ* [*n* = 219] *or -RT* [*n* = 73]) [13], and two cohorts from GEO (*GSE50923-RT/TMZ* [*n* = 49] [14]; *GSE60274-RT/TMZ* [*n* = 32] *or -RT* [*n* = 27] [15]). Moreover, DNA methylation and gene expression data of lower-grade gliomas (LGGs) and nontumor brains from TCGA [16], gliomas of all grades from Chinese Glioma Genome Atlas (CGGA) [17], and nontumor brains from GSE63347 [18] were obtained for additional validation.

Selection and information of all included samples were summarized in Additional file 1: Figure S1 and Additional file 2: Table S1.

### Probe selection and RISK score modeling

**Fig. 1:**
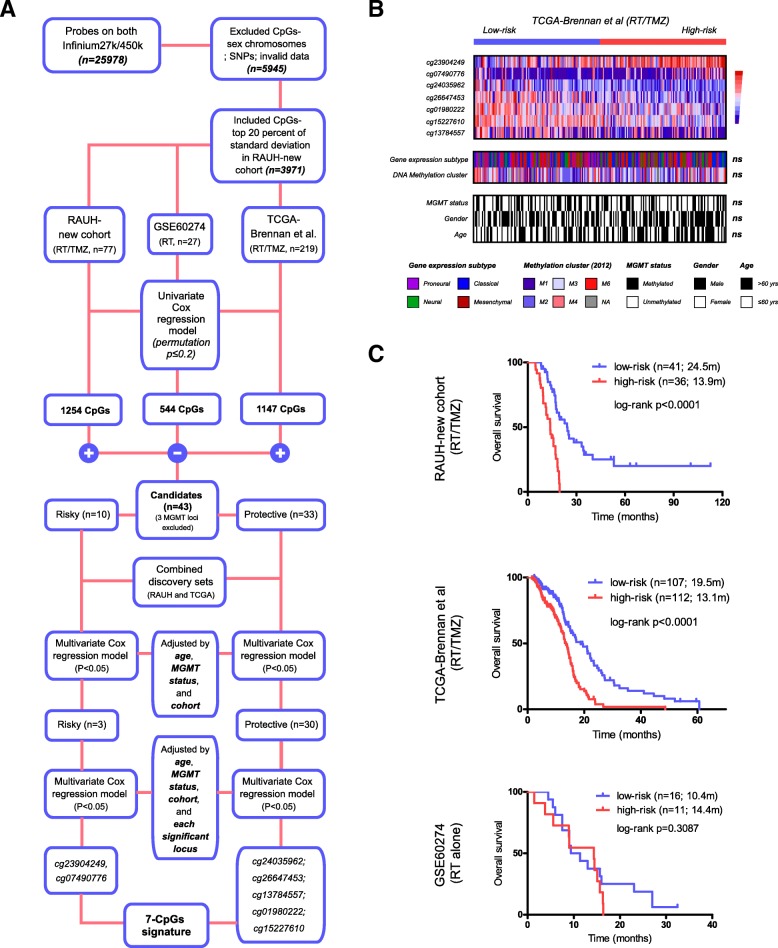
The identification of the 7-CpG signature. **a** The study workflow regarding the selection of predictive epigenetic panel. **b** Molecular and clinical correlations of the multi-CpG signature within *TCGA-Brennan et al.-RT/TMZ*. Heat maps of the methylation patterns of the 7 CpGs are presented, where each row represents a CpG, and each column represents a sample. Clinical and molecular features are indicated for each sample. ns not significant. **c** Patient classification by the 7-CpG RISK score classifier in two discovery cohorts of non-G-CIMP GBMs with RT/TMZ (*RAUH-new cohort* and *TCGA-Brennan et al.*) and one discovery cohort with RT alone (GSE60274);.GBM glioblastoma, G-CIMP glioma-CpGs island methylator phenotype, RT radiotherapy, TMZ temozolomide

Prior probe selection was performed by removal of those not interrogated on both the Infinium HumanMethylation27k and 450k platforms, those targeting the sex chromosomes, and those associated with single-nucleotide polymorphisms. To make DNA methylation microarray data comparable across each dataset, batch effects between each platform and dataset were adjusted by *M*-value transformation and the empirical Bayes approach (*ber* R package) [19, 20]. Missing *β* values were imputed by *impute* R package. Discovery-validation approach was employed for predictive model construction. Two cohorts with RT/TMZ (e.g., *RAUH-new cohort*, *TCGA-Brennan et al-RT/TMZ*) and one cohort with RT alone (e.g., *GSE60274-RT*) were used for discovery phase. Selected CpGs with higher variability in methylation levels (top 20 percent of standard deviation of *β* value) across tumors from *RAUH-new cohort* were used to correlate with overall survival (OS) using univariate Cox regression model and permutation test (Fig. 1a). After removing inconsistent results, an overlap of 43 candidates (permutation *p* < 0.2; excluding three MGMT relevant loci) from the discovery cohorts was subjected to multivariate Cox regression models adjusted by different ages, *MGMT* methylation status, and different cohorts and then to multivariate models incorporating other significant CpGs (Fig. 1a). Finally, a panel of 7 CpGs was identified for constructing a RISK score model (Table 1), which is the sum of *β* values of each CpG weighted by their multivariate Cox coefficients, adjusted by age, MGMT methylation status, patient cohorts, and other loci. The cutoff for low-risk and high-risk tumors were predefined as the median risk score from the combined discovery cohorts with RT/TMZ.

**Table 1:** Characteristics of the seven-CpG panel

Probe ID	Chr.	Relevant gene symbol	Relation to gene region	Relation to CpGs island^b^	Multivariate Cox coefficients^a^
ᅟcg23904249	11	CCDC86	TSS1500	Shore	1.095
cg07490776	8	AP3M2	TSS1500	Island	1.575
cg24035962	10	NCOA4	TSS200	Island	− 1.365
cg26647453	4	C4orf17	5′UTR	Open sea	− 1.574
cg01980222	6	TREM2	1stExon	Open sea	− 1.248
cg15227610	11	HSPB2	TSS1500	Open sea	− 1.120
cg13784557	6	HCP5	TSS200	Open sea	− 0.975

*Chr* chromosome, *TSS* transcriptional start site

^a^Cox coefficients were calculated from multivariate analysis incorporating age, different discovery sets, *MGMT* methylation status, and the seven CpGs within the combined RT/TMZ discovery cohorts

^b^Open sea and shore refers to regions away from relevant CpGs islands more than 4000 bp or less than 2000 bp, respectively

### In vitro functional experiments

The human glioma cell lines T98G, U87, U251, U373, and Hs683 were obtained from American Type Culture Collection (ATCC) and were cultured in Dulbecco’s modified Eagle’s medium supplemented with 10% fetal bovine serum at 37 °C in 5% CO_2_. TMZ (Sigma-Aldrich) was dissolved in dimethyl sulfoxide (DMSO, Sigma-Aldrich) at a stock concentration of 100 mmol/l at 20 °C. Total RNA was purified using TRIzol reagents (Shanghai Pufei Biotechnology) and reverse transcribed with M-MLV RT kit (Promega) [21]. Polymerase chain reaction (PCR) amplification was performed with SYBR Master Mixture (Takara) using LighterCycler 480 II System (Rcoche). The expression values were normalized to the levels of GAPDH. Total DNA was extracted and bisulfate-modified using EZ DNA Methylation-Gold^TM^ Kit (Beijing Tianmo Biotechnology). Pyrosequencing was performed by Pyromark Q96 ID platform and analyzed by PyroMark CpG software (Qiagen). Cell samples were lysed in RIPA buffer [21]. Equal amounts of proteins were resolved by SDS-PAGE gel electrophoresis and transferred to nitrocellulose membranes. The primary antibodies against HSPB2 (Proteintech Group, 21755-1-AP), MGMT (Proteintech Group, 17195-1-AP), and GAPDH (Santa Cruz Biotechnology, SC-32233) were used according to the manufacturers’ recommendations. Each immunoblot was done at least thrice and the signals were quantified using ImageJ software (Bethesda, MD, USA). For HSBP2 *in vitro* overexpression, lentivirus/GV358-HSBP2 (Ubi-HSBP2-3FLAG-SV40-EGFP-IRES-puromycin) and lentivirus/GV358 (control) were obtained from Genechem. Viruses were used to infect GBM cells in the presence of 6 μg/ml polybrene. At 48 to 72 hours after virus infection, puromycin selection (1 μg/ml) was applied and cells without subcloning were used for experiments [21]. CCK-8 kit (Yeasen Inc.) was assayed for cell viability analysis. For 5-Aza-2′-deoxycytidine (5-Aza-dC) demethylation treatment, U251, U87, and U373 cells were grown for 4 days in the presence of DMSO control, 5 μM, and 10 μM 5-Aza-dC (Sigma-Aldrich). Fresh 5-Aza-dC was added every 24 h. For cell apoptosis analysis, TUNEL assay was tested using In Situ Cell Death Detection kit (Roche Diagnostics). Annexin V–fluorescein isothiocyanate/propidium iodide double staining (Roche Diagnostics) was used to sort cells in early or late apoptotic phase.

### Validation cohort of formalin-fixed paraffin-embedded (FFPE) GBM samples

Surgical samples of 333 primary GBMs (Grade IV, WHO) were totally collected from the Department of Neurosurgery, Xijing Hospital, between 2012 and 2016. Inclusion criteria included (1) adult patients (> 18-years old), (2) no prior therapy before surgery, (3) IDH1^R132H^ wild-type tumors, (4) treatment with standard RT plus (adjuvant or concurrent) TMZ or standard RT alone, (5) available OS or progression-free survival (PFS) data, and (6) available FFPE tissue samples. Finally, 54 samples were included for validation analysis, including 32 samples with RT/TMZ and 22 with RT alone. The treatment choices between RT/TMZ and RT were made according to physician’s suggestions and family or patient’s will. Extent of resection was defined by post-operative MRI or CT within one week. The presence of IDH1^R132H^ mutant protein was assessed by immunohistochemistry (IHC) (obtained from the Department of Pathology, Xijing Hospital). FFPE tissues were also employed for IHC with anti-HSPB2 antibody (Proteintech Group, 21755-1-AP). The intensity and percentage of positive cells were evaluated in at least five separate fields at × 400 magnification. The scores were evaluated by two researchers who were blinded to clinical data. Immunoreactivity was scored as follows: 0, no staining; 1, weak staining in < 50% cells; 2, weak staining in w 50% cells; 3, strong staining in < 50%, cells; and 4, strong staining in a 50% cells [21]. Disputes were resolved through discussion. Seven CpGs (74–81) in the promoter region of MGMT and the single CpG (cg155227610) at the non-CpGs island (CGI) region of HSPB2 were detected on the PyroMark Q96ID platform (Qiagen). The average percentage of CGI methylation of 10% was defined as the threshold for unmethylated and methylated MGMT promoter [22]. The median value of HSPB2 single CpG methylation was used as cutoff for hypermethylation and hypomethylation. The primer sequences for PCR and pyrosequencing were listed in Additional file 3: Table S2. All patients provided written informed consent and this study was approved by the Institutional Review Board.

### Statistical analysis

Difference in clinical or molecular features within each risk subgroup was tested by unpaired *t* test, Fisher’s exact, or Chi-square test. Spearman correlation analysis was performed to correlate DNA methylation and gene expression. OS was the time interval from the date of diagnosis or treatment to the date of death or last follow-up. PFS was the time interval from the date of diagnosis or treatment to the date of progression defined by the Macdonald criteria or Response Assessment in Neuro-Oncology (RANO criteria) [23, 24], or the date of death or last follow-up. Survival data was estimated by the Kaplan-Meier Method and compared by log-rank test. Univariate and multivariate Cox regression models were used to evaluate the correlation and independence of each variable. The discriminating ability for prognosis was also evaluated by time-dependent receiver operating characteristic (ROC) curve (*survcomp* R package) [25]. All the calculations were done within SPSS statistics (SPSS Software Inc.) and R software, with two-side *p* values ≤ 0.05 for significance.

## Results

### Identification of a RISK score signature of seven CpGs with potential linkage to TMZ efficacy

According to a strict selection strategy (Fig. 1a), we identified a panel of seven CpGs from the discovery cohorts (Table 1). Each CpG was significantly associated with OS of non-G-CIMP GBMs treated with RT/TMZ, but not RT alone, which was also independent of age, MGMT promoter methylation status, and other CpG members. These 7 CpGs were not among the reported G-CIMP classifiers [9] but seemed to be among the genomic CpGs affected by this molecular status as the CpG panel showed significant but inconsistent alterations in methylation levels among tumors of each G-CIMP status and nontumor brains (Additional file 4: Figure S2A). In addition, the panel appeared not to be correlated with genome hypomethylation, i.e., LINE-1 methylation (Additional file 4: Figure S2A).

The 7-CpG panel was combined using a RISK score model as follows: risk score = (1.095 × *β* value of cg23904249) + (1.575 × *β* value of cg07490776) + (− 1.365 × *β* value of cg24035962) + (− 1.574 × *β* value of cg01980222) + (− 1.248 × *β* value of cg01980222) + (− 1.120 × *β* value of cg15227610) + (− 0.975 × *β* value of cg13784557). Using a predefined cutoff (median risk score, − 1.083), all patients were divided into a low-risk group (with lower risk scores) and a high-risk group (with higher risk scores).

Correlation with known clinical or molecular features in TCGA samples showed that the risk subgroups appeared to not be correlated with gene expression subtypes [26], DNA methylation clusters [13], MGMT promoter methylation status, gender, or age subgroup (Fig. 1b).

By applying the RISK score-based classification to the discovery cohorts, we found that, in *RAUH-new cohort* (RT/TMZ), high-risk patients had shorter OS than low-risk patients (*p* < 0.0001; Fig. 1c). In *TCGA-Brennan et al.-RT/TMZ*, high-risk patients also had poorer OS in comparison with low-risk patients (*p* < 0.0001; Fig. 1c). By contrast, in *GSE60274-RT*, OS was not significantly different between the risk subgroups (*p* = 0.3087; Fig. 1c). The results together suggested a potential linkage of the RISK score signature to TMZ efficacy, instead of a treatment-independent impact on prognosis.

### The prognostic performance of the RISK score signature in validation cohorts

**Fig. 2:**
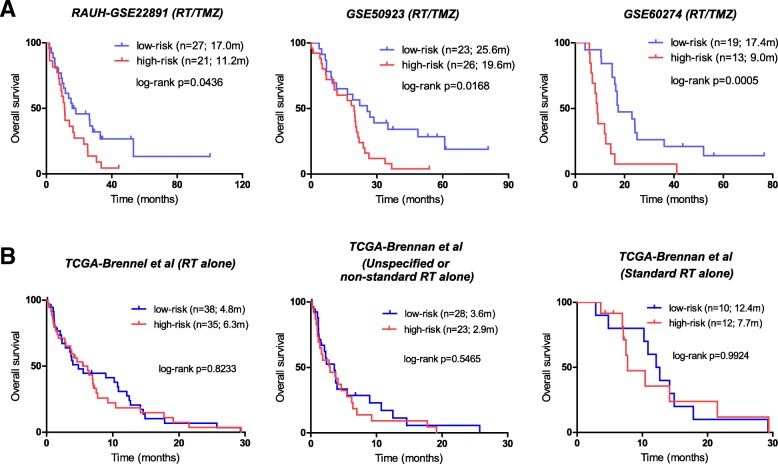
The prognostic performance of the RISK score signature in different validation cohorts of non-G-CIMP GBMs, **a** Patient classification in validation cohorts with the combination of RT and TMZ, **b** Patient classification in validation cohorts with RT alone. GBM glioblastoma, G-CIMP glioma-CpGs island methylator phenotype, RT radiotherapy, TMZ temozolomide

To further investigate the prognostic performance of the RISK score signature, we then applied it to a series of validation cohorts with different treatments. The signature accurately predicted OS in the validation cohorts with RT/TMZ: *RAUH-GSE22891* (*p* = 0.0436), *GSE50923-RT/TMZ* (*p* = 0.0168), and *GSE60274-RT/TMZ* (*p* = 0.0005; Fig. 2a). The signature also predicted PFS in two available cohorts with RT/TMZ: *RAUH-new cohort* (*p* < 0.0001) and *TCGA-Brennan et al.-RT/TMZ* (*p* = 0.0151; Additional file 5: Figure S3A). However, the signature was unable to predict OS in a validation cohort with RT alone, i.e., *TCGA-Brennan et al.-RT* (Fig. 2b). Cox regression analyses of *RAUH-new cohort* and *TCGA-Brennan et al.-RT/TMZ* confirmed the RISK score signature as a significant risk factor that is independent of MGMT methylation status, as well as other variables (e.g., age, treatment schedules, treatment at progression), among non-G-CIMP GBMs with RT/TMZ (Additional file 6: Table S3), instead of RT alone (Additional file 7: Table S4). Those findings indicate that the RISK score signature might not be a general prognostic biomarker that is independent of treatment, but rather has a specific linkage to TMZ efficacy in non-G-CIMP GBMs.

### The RISK score signature might be a promising predictive indicator of TMZ response

**Fig. 3:**
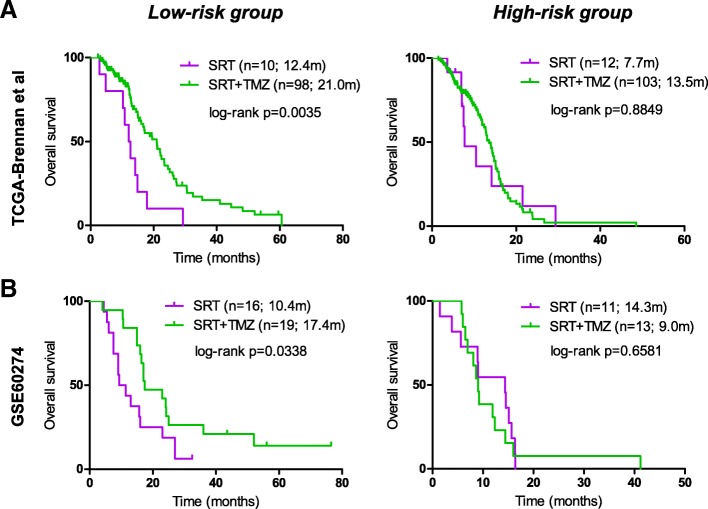
The predictive performance of the RISK score signature in different validation cohorts. Interaction analysis between treatments (with versus without TMZ) and risk subgroups (low-risk versus high-risk) in **a** TCGA-Brennan et al. and **b** GSE60274. Only patients with standard RT regimen were included for analysis in order to reduce potential bias by heterogeneous treatment regimen. RT radiotherapy, TMZ temozolomide

To investigate whether the RISK score signature has a predictive ability for TMZ response, interaction analyses were carried out between the risk subgroups and treatments, incorporating only patients with standard RT alone or combined with (concurrent or adjuvant) TMZ from *TCGA-Brennan et al.* and *GSE60274*. No significant difference was observed in baseline information (e.g., age, pre-adjuvant KPS, gender) between patients with different treatments in each risk subgroup (data not shown). The interaction analyses showed that standard RT/TMZ did confer a clear OS benefit to low-risk patients compared to standard RT, and this treatment was associated with a similar OS in high-risk patients (Fig. 3). Similar results were observed in terms of PFS (Additional file 8: Figure S4). Cox regression analyses of *TCGA-Brennan et al.* and *GSE60274* confirmed standard RT/TMZ as a favorable indicator for OS benefit, independent of MGMT methylation status and age, in low-risk patients (Additional file 9: Table S5), but not in high-risk patients (Additional file 10: Table S6). Together, those results indicate that the RISK score signature might be a potential predictive indicator of TMZ outcome and be helpful for identifying subpopulations of patients who are likely to benefit from TMZ treatment. These findings should be conservatively interpreted due to the potential patient bias during treatment assignment in a retrospective series.

### Patient classification in stratified cohorts by MGMT promoter methylation status and age

**Fig. 4:**
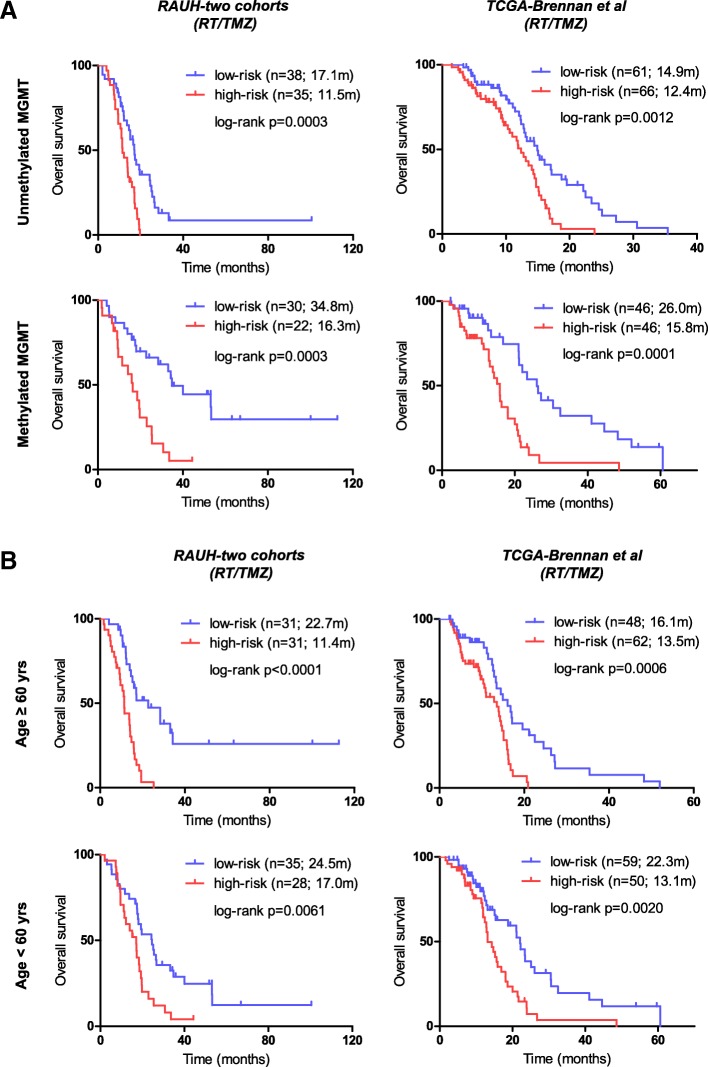
The patient classification of the RISK score signature in cohorts stratified by conventional risk factors. **a** Cohorts stratified by MGMT promoter methylation status. **b** Cohorts stratified by patient age (≥ 60 vs. < 60 yrs). MGMT O-6-methylguanine-DNA methyltransferase, RT radiotherapy, TMZ temozolomide

To further explore the clinical impact of the epigenetic signature, we also evaluated its performance in cohorts stratified by MGMT methylation status and age. We found that the RISK score signature showed significant discriminating value for OS of patients with each MGMT methylation status in the combined RAUH cohorts (*RAUH-two cohorts*) and in *TCGA-Brennan et al.-RT/TMZ* (Fig. 4a). Similarly, the signature robustly predicted OS in each age subgroup (age < vs. ≥ 60 years old; Fig. 4b). The results were also observed in two GEO cohorts (Additional file 11: Figure S5A-B). The combination of the RISK score signature with the two conventional risk factors could provide optimized risk classification in non-G-CIMP GBMs (Additional file 12: Figure S6A-B). Moreover, for those who underwent RT/TMZ, the time-dependent ROC showed that the RISK score signature was superior to MGMT methylation status in predicting OS among older patients (≥ 60 years) but had less discriminating value among younger patients (Additional file 12: Figure S6C).

### HSPB2 appeared to be epigenetically regulated by non-CGI methylation and was associated with TMZ resistance in GBM cell lines

**Fig. 5:**
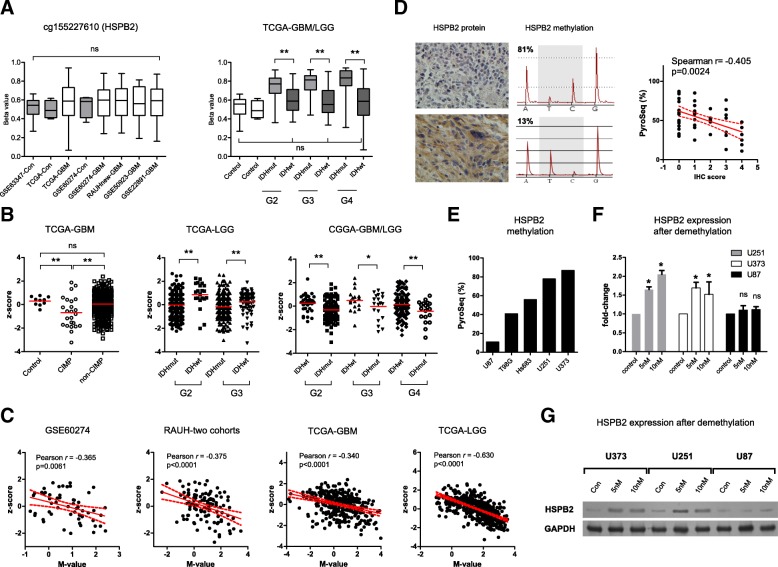
DNA methylation and gene expression of HSPB2. **a** The single CpG methylation of HSPB2 among nontumor brains and GBMs or gliomas of each grade. **b** gene expression patterns of HSPB2 among nontumor brains and GBMs or gliomas of each grade. **c** Correlations of the single CpG methylation and gene expression of HSPB2 in each cohort. **d** Representative HSPB2 IHC staining and pyrosequencing results (left) and the correlation of IHC staining scores and pyrosequencing data (right) in FFPE samples of IDH^R132H^ wild-type GBMs. **e** HSPB2 single CpG pyrosequencing data in each GBM cell line. **f**, **g** Real-time PCR and western blot results of HSPB2 expressions in different GBM cell lines with either hypermethylated CpG (e.g., U251 and U373) or hypomethylated CpG (e.g., U87) after demethylation treatment with 5-Aza-dC at different doses (e.g., control, 5 nM, 10 nM). GBM glioblastoma, G-CIMP glioma-CpGs island methylator phenotype, FFPE formalin-fixed paraffin-embedded, wt wild type

**Fig. 6:**
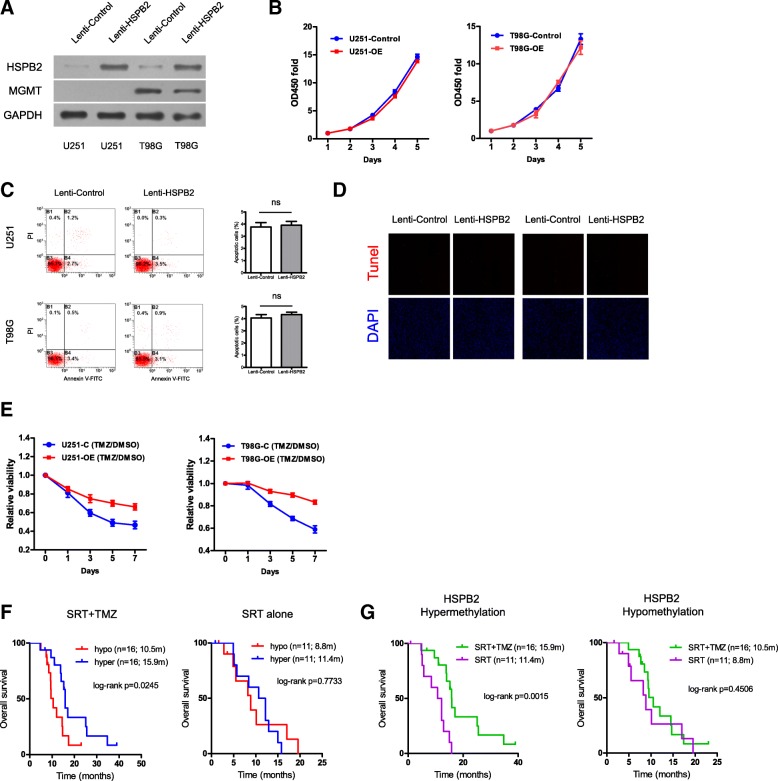
The impacts of HSPB2 methylation and expression on TMZ resistance. **a** HSPB2 overexpression using lentivirus infections was confirmed by western blot in U251 and T98G cells regardless of MGMT expression. **b** CCK-8 proliferation assays of U251 and T98G cells infected with lentivirus containing human *HSPB2* ORF or empty control lentivirus. **c**, **d** Flow cytometry and TUNEL assay analysis detecting apoptotic cells in U251 and T98G cells after infections with lentivirus containing human *HSPB2* ORF or empty control lentivirus. **e** CCK-8 assays of U251 and T98G cells with or without HSPB2 overexpression after the treatment of TMZ at 100 μM or an equal volume of DMSO for 7 consecutive days. **f** The single CpG methylation of HSPB2 by pyrosequencing predicted OS in FFPE samples of IDH1^R132H^ wild-type GBMs treated with the combination of RT and TMZ (left) but not in those with RT alone (right). **g** Interaction analysis showed that standard RT plus TMZ conferred a clear OS benefit to patients with hypermethylated HSPB2, rather than those with hypomethylated HSPB2, in comparison with standard RT alone. TMZ temozolomide, ORF open reading frame, RT radiotherapy, DMSO dimethyl sulfoxide, OS overall survival, FFPE formalin-fixed paraffin-embedded, GBM glioblastoma

To gain biological insight into the multimarker epigenetic signature, we selected one of the 7 CpGs (cg15227610) for further analysis (Table 1). This single CpG was located at the non-CGI transcriptional regulatory region of HSPB2. The single CpG methylation and HSPB2 expression differed regarding the G-CIMP status (or IDH mutations), but they were not significantly altered in GBMs or in IDH mutant gliomas compared to nontumor brains (Fig. 5a, b). The status of single CpG methylation and HSPB2 expression did not differ across tumors of different grades (Fig. 5b). Notably, the single CpG methylation was consistently and significantly negatively correlated with HSPB2 expression (Fig. 5c). The negative correlation between methylation and protein levels was also observed in a local cohort of FFPE samples (Fig. 5d). Demethylation treatment with 5-Aza-dC showed that HSBP2 expression was increased in GBM cells that had the original hypermethylated CpG, e.g., U373 and U251, but not in cells with hypomethylated CpG, e.g., U87 (Fig. 5e, g). Considering that HSPB2 expression is relatively low in GBM cell lines (data not shown), HSPB2 overexpression by lentivirus infections was conducted and confirmed by western blot (Fig. 6a) and was not associated with significant alterations in proliferation and apoptosis (Fig. 6b–d). However, HSPB2 overexpression did confer resistance to TMZ treatment in GBM cells regardless of MGMT expression (Fig. 6e).

### Clinical performance of the single CpG methylation of HSPB2 by pyrosequencing in an IDH1^R132H^ wild-type GBM cohort of FFPE samples

The validation cohort of 54 IDH1^R132H^ wild-type GBMs was associated with similar patient age, gender, and pre-adjuvant therapy KPS compared to all collected IDH1^R132H^ wild-type samples from our center (Additional file 1: Figure S1). Similarly, the clinical characteristics appeared to be similar between those patients who received RT/TMZ versus RT alone (Additional file 2: Table S1). The single CpG methylation of HSPB2 by pyrosequencing predicted OS in patients who received RT/TMZ (log-rank *p* = 0.0245) but not in those who received RT alone (log-rank *p* = 0.7733; Fig. 6f). Despite not reaching significance, similar findings were observed for PFS outcome (Additional file 5: Figure S3B). Interaction analyses and Cox analyses both supported an independent predictive potential of the single CpG methylation of HSPB2 to TMZ efficacy in clinically available FFPE samples (Fig. 6g, Additional file 8: Figure S4B and Additional file 9: Table S5 and Additional file 10: Table S6). Moreover, HSPB2 methylation could optimize the risk classification by MGMT promoter methylation status (Additional file 11: Figure S5C).

## Discussion

Clinically informative biomarkers predictive of the likely benefit from specific treatments are of great significance in guiding precision medicine in cancer patients [27]. Epigenetic marks and DNA methylation in particular have long been regarded as the leading candidates for biomarker discovery as they have many advantages over genetic or expression-based information such as having reliable DNA samples, altered patterns that have stability, tolerance of nontumor cell contamination, multilevel biological relevance, and drug-induced reversibility [28, 29]. The promoter methylation status of MGMT for predicting TMZ outcome is considered an example of using DNA methylation as a more powerful indicator compared to other molecular information [30]. Unfortunately, this single-gene epigenetic status has critical blind spots regarding guiding treatment decisions for the very heterogeneous groups of GBM patients.[8, 31] Therefore, the development of powerful predictive indicator that could take advantage of multimarker information and provide complementary information to MGMT promoter methylation status would be greatly helpful for improving current clinical decision-making.

In this study, the Illumina 450k array provided a dramatic increase in the genomic coverage of CpGs compared to the 27k array. However, only a limited number of samples were available with Illumina 450k data. To ensure that there were enough samples to employ a discovery-validation approach, we decided to limit CpGs to those that appeared both on the 27k and 450k arrays, at the expense of higher genomic coverage. Regarding the outcome measurements, we choose OS (rather than PFS) as the primary endpoint for observation of the clinical benefit of TMZ. OS had been reported to be a reasonable endpoint to measure the clinical benefit of a given therapy, especially for advanced diseases with poor prognoses [32]. Additional considerations were the inconsistent assessment criteria for tumor progression and the unavailability of PFS data in the included cohorts. We employed a strict selection strategy to search novel CpGs with potential predictive value for TMZ outcome; the approach appeared to be efficient, as it could also identify MGMT-related loci. Following this strategy, we identified a panel of 7 CpGs from the discovery cohorts of RT/TMZ versus RT alone, each of which predicted OS of non-G-CIMP GBM patients, independent of their MGMT methylation status, age, and other identified CpGs. To coordinate these 7 CpGs, a RISKscore algorithm was used to produce a multimarker signature. Applying the signature to different validation cohorts with either RT/TMZ or RT alone showed that it was not a general prognostic biomarker for non-G-CIMP GBMs, as a prognostic factor is commonly defined as a clinical or biological characteristic that provides information on the likely outcome of a disease regardless of treatment [8, 33]. In contrast, a predictive factor is to provide information on the likely response to treatment and is used to identify subpopulations of patients who are most likely to benefit from a given therapy [8, 33]. Accordingly, the interaction analyses between the risk subgroups and treatments found a promising predictive value of the RISK score signature for the efficacy of TMZ, as the signature could identify the subgroups of low-risk patients who appeared to benefit more from RT/TMZ compared to RT alone. Thus, we concluded that the 7-CpG signature might not be a general treatment-independent prognostic biomarker but rather a promising predictive indicator of TMZ response in non-G-CIMP GBMs. These conclusions should be conservatively interpreted due to the following study limitations: (1) the prognostic effect has not been finally validated in patients who received no adjuvant therapies [33], and (2) the predictive effect has not been retrospectively or prospectively verified in a randomized controlled trial (RCT) [33]. Other limitations also confounded our results such as small sample size especially for patients who underwent only RT, potential biases in treatment assignment and patient baseline characteristics, heterogeneous treatment regimens, and insufficient clinical information (e.g., PFS, corticosteroid usage, regimens and salvage treatment). In addition, G-CIMP GBMs were excluded from our analyses because those tumors, exclusively carrying IDH mutations, represented a small subtype (~ 10% of all GBMs) and showed a very distinct molecular background and clinical prognosis compared to non-G-CIMP GBMs [9, 16].

The promoter methylation status of MGMT has been by far the most informative biomarker for GBMs; however, mandatory testing for this is highly controversial due to insufficient evidence showing a direct relationship between MGMT testing and TMZ usage [8]. Previous data has shown that despite with the much reduced benefits, the combination of RT and TMZ could still confer a significant improvement in OS among patients with unmethylated tumors, especially in younger patients [7]. Considering the absence of effective alternative therapies and a generally good tolerance to the aggressive combined treatment, TMZ is unlikely to be withheld from standard care [8]. The clinical value of MGMT testing is thus much compromised. In this study, we found that the RISK score signature might provide complementary information to MGMT for predicting TMZ outcome as it showed good and consistent discriminating value for the prognosis of non-G-CIMP tumors, independent of MGMT methylation status. The RISK score signature might be helpful for refining current TMZ usage practices by identifying patients with unmethylated tumors who might also benefit from TMZ, while sparing those who might not benefit from the high cost and potential toxicity related to this treatment. Though encouraging, the findings of this study should be fully validated in a RCT. The application of the combined RT/TMZ is complicated for elderly GBM patients [34]. Many physicians are still reluctant to treat elderly patients as aggressively as younger patients, citing concerns about overall poor physical condition, the presence of comorbidities, and a decreased tolerance to effective therapies. An increasing number of studies have reported clear benefits of RT/TMZ in elderly patients [34, 35]. However they all have highlighted the importance of appropriate patient selection for the optimal usage of the aggressive therapy in this frail population [34]. In addition to clinical factors (e.g., extent of resection, KPS, and comprehensive geriatric assessment), molecular data may also provide useful information [35]. Two recent phase III randomized trials have demonstrated a direct linkage between MGMT testing and TMZ usage in elderly patients [36, 37]. In our study, the RISK score signature showed good discriminating value for the prognosis of patients with different ages and even had a superior predictive ability compared to MGMT methylation status in older populations. Overall, the RISK score signature could be of potential use for individualized therapy by aiding in the appropriate patient selection and optimizing the current risk classification in GBMs.

The biological implications of the multimarker signature were exemplified by one CpG component and its relevant gene, HSPB2. HSPB2 is a member of the small heat shock protein gene family, encoding a molecular chaperone, and usually plays a protective role for cells from deleterious stresses such oxidative stress, heat shock, radiation and toxic drugs [38]. HSPB2 has been reported to be epigenetically or transcriptionally altered in some human cancers, with potential roles in tumor growth, metastasis, and in particular drug resistance [39]. In this study, the single CpG methylation was found to be negatively correlated with HSPB2 expression, indicating an epigenetic regulatory mechanism for this gene. The DNA methylation status and gene expression of HSPB2 were not significantly altered between nontumor brains and non-G-CIMP/IDHwt gliomas of each grade, indicating that it is not a good candidate for diagnostic use. In vitro experimental data showed that HSPB2 overexpression did not affect cell proliferation and apoptosis in GBM cells but conferred resistance to TMZ treatment regardless of MGMT expression. Together, these data may provide some biological explanations regarding the predictive effects of the multimarker signature—the epigenetic panel might contribute to TMZ resistance via epigenetically regulating the expression levels of drug-resistant genes. Genes (or RNAs) related to other identified CpGs have also been reported to have implications in cancers such as the long non-coding (lnc) RNA HCP5 and the gene TREM2 in gliomas [40, 41], and the genes NCOA4 and C4orf17 in prostate and ovarian cancers[42–44]. Notably, among the 7-CpG panel, some CpGs did not show a significant correlation between DNA methylation and the corresponding gene expression (data not shown). The biological impacts of those epigenetic alterations on TMZ resistance remained largely unclear. Recent studies reported that in addition to a direct alteration in gene expression, cancer-linked DNA methylation abnormalities may have functional impacts via contributing to disrupted heterochromatin, leading to loss of both epigenetic and transcriptional regulatory mechanisms and resulting in hypervariable expression [45]. Future studies will be needed to explore the biological relevance of other identified CpGs and the specific molecular mechanism of HSBP2-mediated TMZ resistance.

Unfortunately, our microarray-based signature is not available for clinical use due to the inconvenience and unavailability of the genome-wide detection technique in real clinical setting. Quantitative pyrosequencing is a well-established and widely used method for single CpG methylation detection. A comparative study has showed excellent congruence of DNA methylation data from the Illumina DNA methylation array with pyrosequencing data [46]. Therefore, the conventional technique of pyrosequencing may represent a good alternative solution for the 7-CpG profiling. Validation of the predictive ability of the single CpG methylation of HSPB2 by pyrosequencing in clinically available FFPE samples was reported not only to provide additional validation of the multi-CpG signature at a clinical level, but also to highlight the promising translational potential of the array-based signature to routine clinical testing. Independent validation studies will, however, be needed for adjusting the microarray-based signature to a pyrosequencing-based signature because there are inconsistencies between the two methods in the DNA methylation data for individual loci [46].

Recent multiplatform molecular profiling analysis had revealed that LGGs with wide-type IDH and intact 1p/19q have remarkable genomic and clinical similarity to primary non-G-CIMP GBMs [16]. Such similarity supports the potential inclusion of this subtype of LGGs within the broad spectrum of GBM-related clinical investigation and biomarker validation [16]. In this study, we have validated that the GBM-derived epigenetic signature could predict OS in an RT/TMZ cohort of GBM-like LGGs without IDH mutations and 1p/19q codeletions from TCGA (unknown doses of RT and TMZ, *p* = 0.0077; Additional file 13: Figure S7), its predictive performance however should be further investigated in this LGGs subtype without TMZ therapy.

In summary, we proposed a RISK score signature comprising 7 CpGs and the single CpG methylation of HSPB2, both with promising predictive values for the outcome of TMZ therapy in non-G-CIMP (or wild-type IDH) GBMs. These predictors might also provide complementary information to the current best MGMT-based predictor. Future prospective studies are needed to definitively establish these conclusions.

## Additional files

Additional file 1: **Figure S1.** The diagram of patient selection in each cohort; (A) selection of non-G-CIMP GBMs with RT/TMZ or RT alone; patient characteristics of included and excluded samples from TCGA-Brennan et al. were compared and presented; (B) selection of IDHwt+1p/19q-intact LGGs with RT/TMZ; patient characteristics of included and excluded samples from TCGA-LGGs were compared and presented; (C) selection of IDH1^R132H^ wild-type GBMs with RT/TMZ or RT alone; patient characteristics of included and excluded FFPE samples from Xijing hospital were compared and presented; RT=radiotherapy; TMZ=temozolomide; G-CIMP=glioma CpG island methylator phenotype; GBM=glioblastoma; LGG=lower-grade gliomas; TCGA=The Cancer Genome Atlas; FFPE= formalin-fixed paraffin-embedded; OS=overall survival; M=male; F=female. (PDF 342 kb)

Additional file 2: **Table S1.** Sample information of discovery and validation cohorts. (XLSX 16 kb)

Additional file 3: **Table S2.** The primer sequences for PCR and pyrosequencing. (XLSX 13 kb)

Additional file 4: **Figure S2.** The molecular correlation of each CpGs with genome DNA methylation status in GBMs; (A) the methylation status of each CpGs and the RISK scores between non-tumor brains, G-CIMP and non-G-CIMP tumors; (B) the correlation of the methylation status of each CpGs and the RISK scores with LINE-1 methylation; non-tumor brains from GSE63347 and TCGA as controls; * and ** indicates *P* <0.01 and <0.001; GBM=glioblastoma; G-CIMP=glioma CpG island methylator phenotype; TCGA=The Cancer Genome Atlas; ns=non-significance (*p* >0.05). (PDF 956 kb)

Additional file 5: **Figure S3.** The prognostic performance in terms of PFS outcome; (A) patient classification by the RISK-score signature in RAUH-new cohort (*left*) and TCGA-Brennan et al. (*right*); (B) patient classification by HSPB2 methylation pyrosequencing in validation cohort of FFPE samples with RT/TMZ (*left*) and RT alone (*right*); PFS=progression-free survival; RT=radiotherapy; TMZ=temozolomide; RAUH=Rennes and Angers University Hospitals; FFPE=formalin-fixed paraffin-embedded. (PDF 424 kb)

Additional file 6: **Table S3.** Univariate and multivariate Cox regression analyses in non-G-CIMP/IDH1R132H wt GBMs with RT/TMZ. (XLSX 27 kb)

Additional file 7: **Table S4.** Univariate and multivariate Cox regression analyses in non-G-CIMP/IDH1R132H wt GBMs with RT alone. (XLSX 25 kb)

Additional file 8: **Figure S4.** The predictive performance in terms of PFS outcome; (A) interaction analysis between treatments (with versus without TMZ) and risk subgroups (low-risk versus high-risk) in *TCGA-Brennan et al*, and (B) interaction analysis between treatments (with versus without TMZ) and risk subgroups (HSPB2 hypomethylation versus hypermethylation) in Xijing cohort; only patients with standard RT regimen were included for analysis in order to reduce potential bias by heterogeneous treatment regimen; SRT= standard radiotherapy; TMZ=temozolomide. (PDF 399 kb)

Additional file 9: **Table S5.** Univariate and multivariate Cox regression analyses in low-risk group of non-G-CIMP (or HSPB2 hypermethylated IDH1R132H wt) GBMs. (XLSX 23 kb)

Additional file 10: **Table S6.** Univariate and multivariate Cox regression analyses in high-risk group of non-G-CIMP (or HSPB2 hypomethylated IDH1R132H wt) GBMs. (XLSX 23 kb)

Additional file 11: **Figure S5.** The patient classification in cohorts stratified by MGMT promoter methylation status and age; The RISK-score signature in the pooled GEO cohorts with RT/TMZ (GSE50923 and GSE60274 collectively) having (A) MGMT unmethylated (*left*) and methylated tumors (*right*) or (B) having older age (*left*) and younger age (*right*); HSPB2 methylation in Xijing cohort of FFPE tissues with RT/TMZ with MGMT unmethylated (*left*) and methylated tumors (*right*); GEO=Gene Expression Omnibus; FFPE=formalin-fixed paraffin-embedded; RT=radiotherapy; TMZ=temozolomide; MGMT=O-6-methylguanine-DNA methyltransferase. (PDF 508 kb)

Additional file 12: **Figure S6.** The clinical performance of the RISK-score signature in combination with conventional risk factors in the setting of the combination treatment of RT and TMZ; (A) the risk classification of the RISK-score signature in combination with age (≥60 vs. <60 yrs; *left*) and MGMT promoter methylation status (*right*); (B) time-dependent ROC values of the RISK-score signature, age, MGMT promoter methylation status, and their combinations at each time point within all patients who underwent RT/TMZ from RAUH and TCGA-Brennan et al; (C) time-dependent ROC values of the RISK-score signature in comparison with MGMT promoter methylation status in subgroups of different ages at each time point; TCGA=The Cancer Genome Atlas; RAUH=Rennes and Angers University Hospitals; RT=radiotherapy; TMZ=temozolomide; MGMT=O-6-methylguanine-DNA methyltransferase; yrs=years. (PDF 749 kb)

Additional file 13: **Figure S7.** The prognostic performance of the GBM-derived epigenetic signature in a RT/TMZ cohort of GBM-like LGGs without *IDH* mutations and 1p/19q co-deletion from TCGA; TCGA=The Cancer Genome Atlas; GBM=glioblastoma; RT=radiotherapy; TMZ=temozolomide; LGGs=lower-grade glioma; IDH= isocitrate dehydrogenase. (PDF 227 kb)

## Abbreviation

ATCC: American Type Culture Collection
CI: Confidence interval
DMSO: Dimethyl sulfoxide
FFPE: Formalin-fixed paraffin-embedded
GBM: Glioblastoma
G-CIMP: Glioma-CpGs island methylator phenotype
HR: Hazard ratios
IDH: Isocitrate dehydrogenase
IHC: Immunohistochemistry
lncRNA: Long non-coding RNA
KPS: Karnofsky performance score
LGG: Lower-grade glioma
MGMT: O-6-methylguanine-DNA methyltransferase
ORF: Open reading frame
OS: Overall survival
PCR: Polymerase Chain Reaction
RAUH: Rennes and Angers University Hospitals
RCT: Randomized controlled trial
ROC: Receiver operating characteristic
RT: Radiotherapy
TCGA: The Cancer Genome Atlas
TMZ: Temozolomide
