# Supplementary figures and images for: Novel predictive epigenetic signature for temozolomide in non-G-CIMP glioblastomas
Source: Clin Epigenetics. 2019 May 14;11:76. doi: 10.1186/s13148-019-0670-9 (PMC6515684; doi:10.1186/s13148-019-0670-9)

A

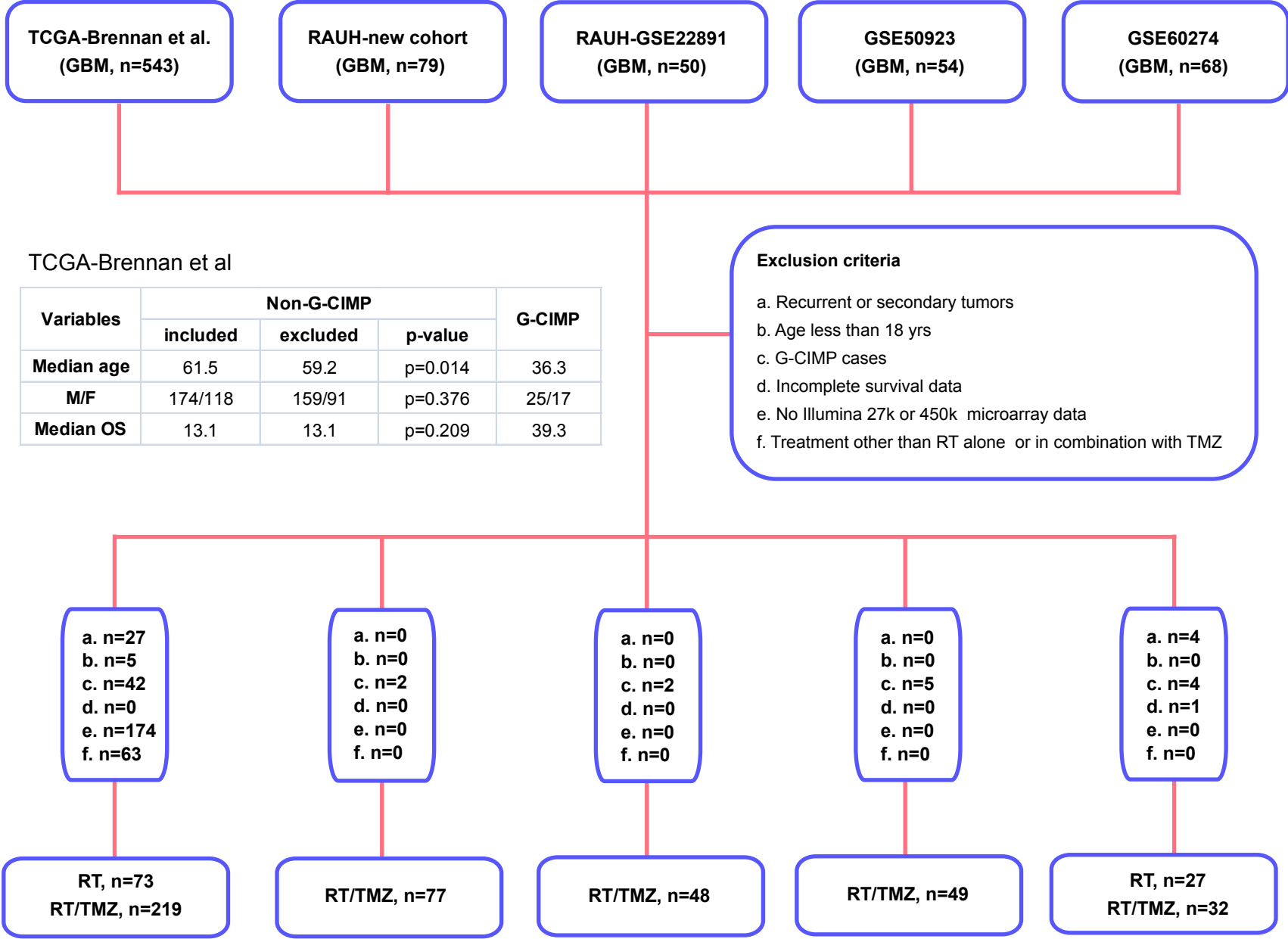

B

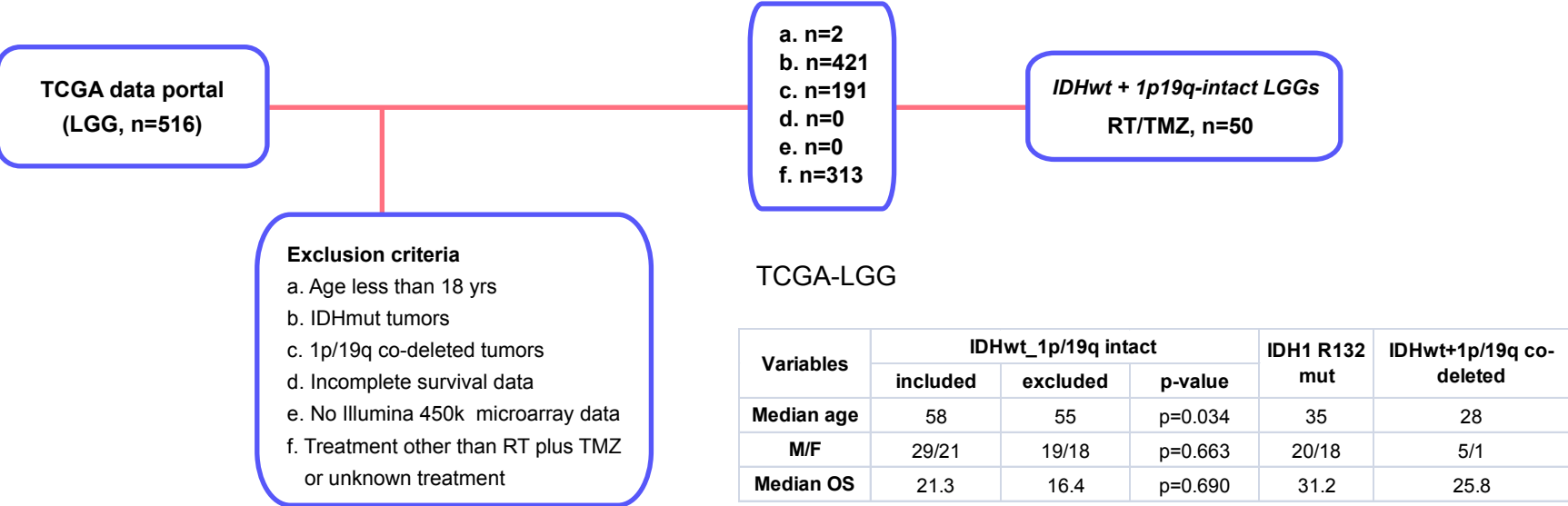

C

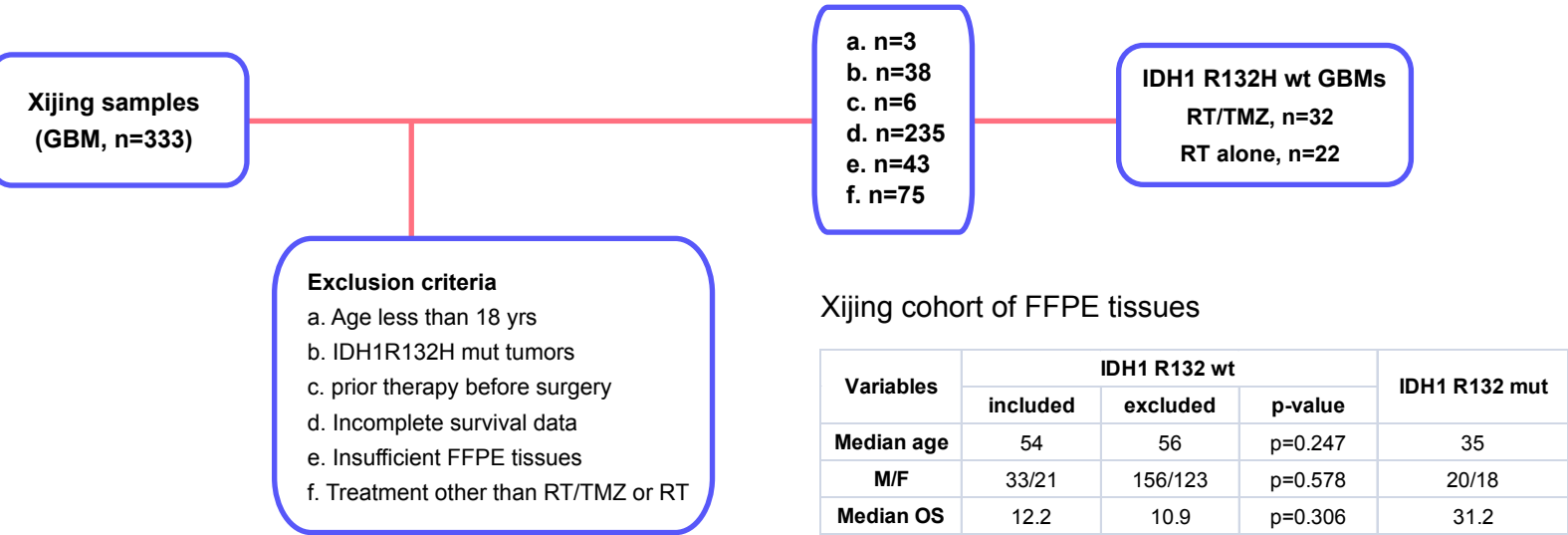

Supplement: Supplementary file 1 — Figure S1. The diagram of patient selection in each cohort; (A) selection of non-G-CIMP GBMs with RT/TMZ or RT alone; patient characteristics of included and excluded samples from TCGA-Brennan et al. were compared and presented; (B) selection of IDHwt+1p/19q-intact LGGs with RT/TMZ; patient characteristics of included and excluded samples from TCGA-LGGs were compared and presented; (C) selection of IDH1R132H wild-type GBMs with RT/TMZ or RT alone; patient characteristics of included and excluded FFPE samples from Xijing hospital were compared and presented; RT=radiotherapy; TMZ=temozolomide; G-CIMP=glioma CpG island methylator phenotype; GBM=glioblastoma; LGG=lower-grade gliomas; TCGA=The Cancer Genome Atlas; FFPE= formalin-fixed paraffin-embedded; OS=overall survival; M=male; F=female. (PDF 342 kb) [file 13148_2019_670_MOESM1_ESM.pdf]

A

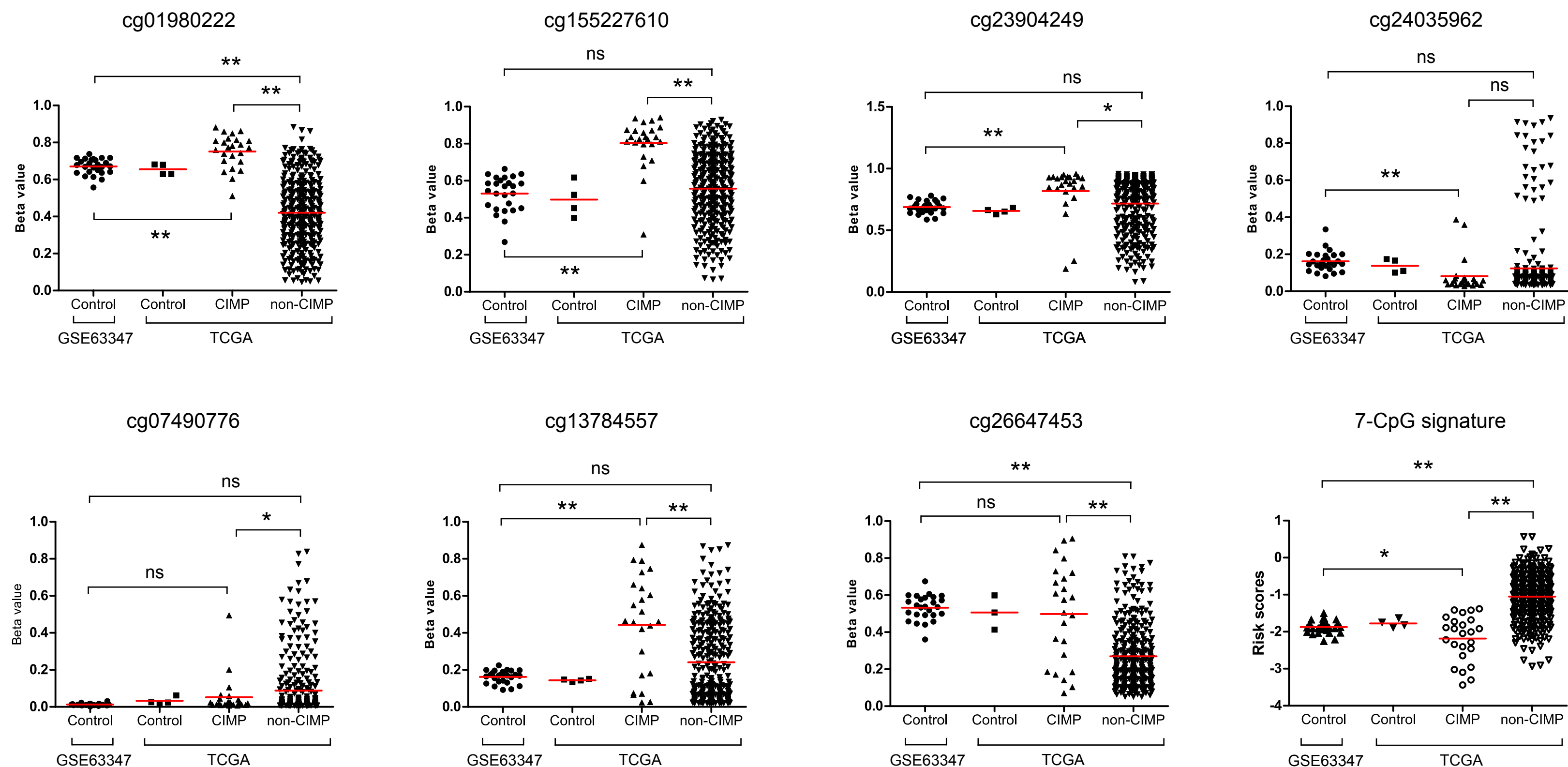

B

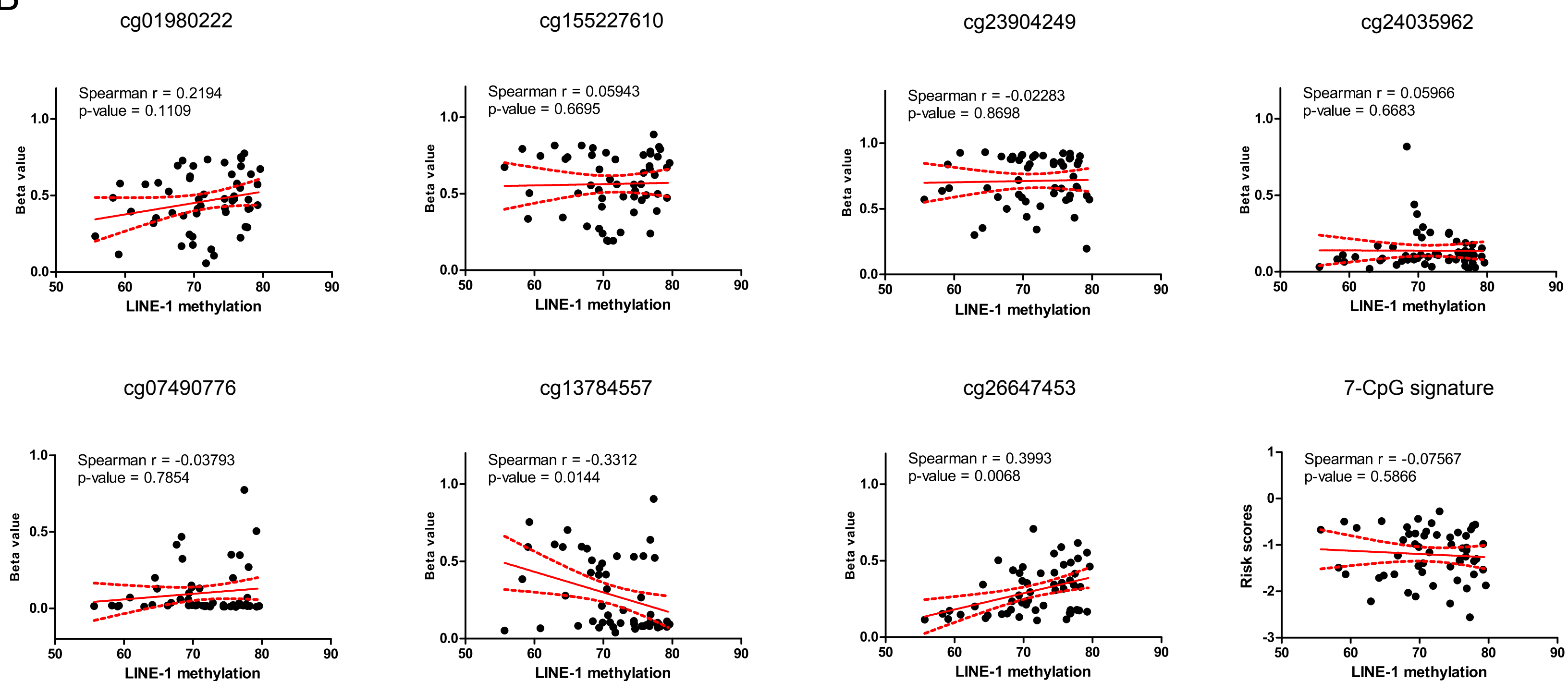

Supplement: Supplementary file 4 — Figure S2. The molecular correlation of each CpGs with genome DNA methylation status in GBMs; (A) the methylation status of each CpGs and the RISK scores between non-tumor brains, G-CIMP and non-G-CIMP tumors; (B) the correlation of the methylation status of each CpGs and the RISK scores with LINE-1 methylation; non-tumor brains from GSE63347 and TCGA as controls; * and ** indicates P <0.01 and <0.001; GBM=glioblastoma; G-CIMP=glioma CpG island methylator phenotype; TCGA=The Cancer Genome Atlas; ns=non-significance (p >0.05). (PDF 956 kb) [file 13148_2019_670_MOESM4_ESM.pdf]

**A*****RAUH-new cohort (RT/TMZ)***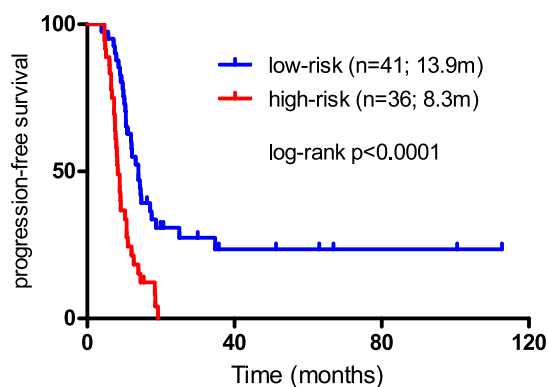***TCGA-Brennan et al (RT/TMZ)***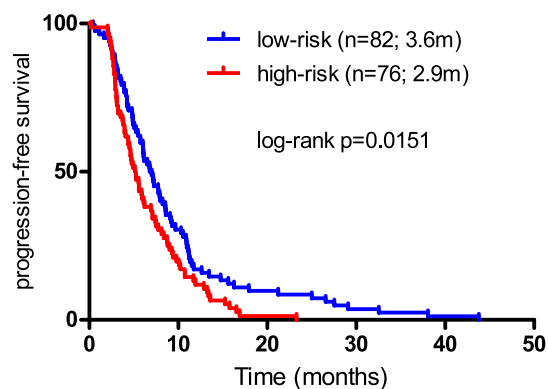**B*****FFPE cohort (RT/TMZ)***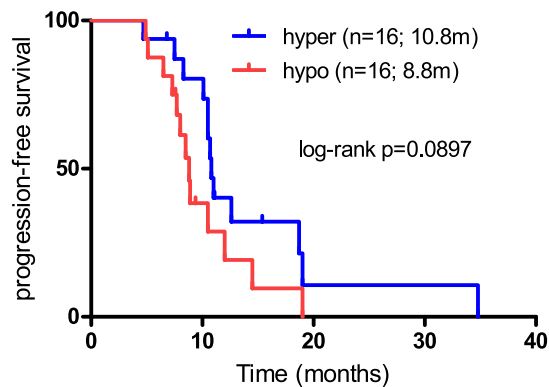***FFPE cohort (RT alone)***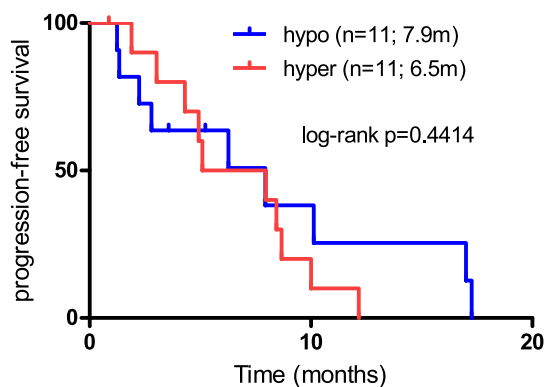

Supplement: Supplementary file 5 — Figure S3. The prognostic performance in terms of PFS outcome; (A) patient classification by the RISK-score signature in RAUH-new cohort (left) and TCGA-Brennan et al. (right); (B) patient classification by HSPB2 methylation pyrosequencing in validation cohort of FFPE samples with RT/TMZ (left) and RT alone (right); PFS=progression-free survival; RT=radiotherapy; TMZ=temozolomide; RAUH=Rennes and Angers University Hospitals; FFPE=formalin-fixed paraffin-embedded. (PDF 424 kb) [file 13148_2019_670_MOESM5_ESM.pdf]

**A**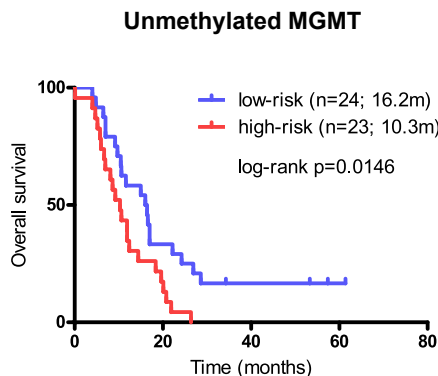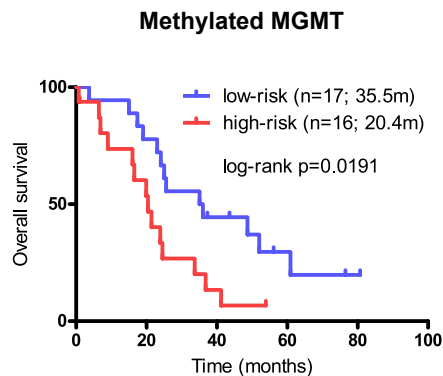**B**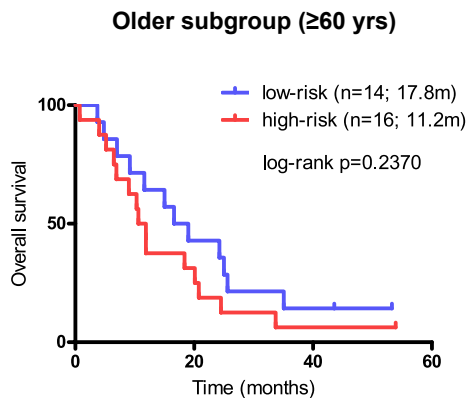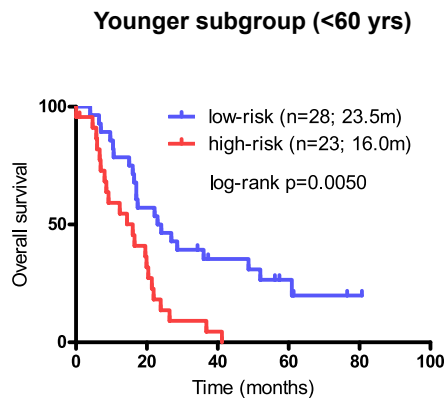**C**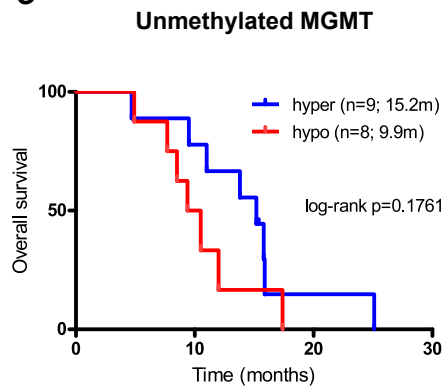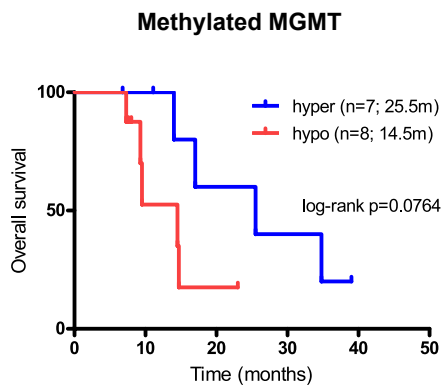

Supplement: Supplementary file 11 — Figure S5. The patient classification in cohorts stratified by MGMT promoter methylation status and age; The RISK-score signature in the pooled GEO cohorts with RT/TMZ (GSE50923 and GSE60274 collectively) having (A) MGMT unmethylated (left) and methylated tumors (right) or (B) having older age (left) and younger age (right); HSPB2 methylation in Xijing cohort of FFPE tissues with RT/TMZ with MGMT unmethylated (left) and methylated tumors (right); GEO=Gene Expression Omnibus; FFPE=formalin-fixed paraffin-embedded; RT=radiotherapy; TMZ=temozolomide; MGMT=O-6-methylguanine-DNA methyltransferase. (PDF 508 kb) [file 13148_2019_670_MOESM11_ESM.pdf]

***TCGA-LGGs (RT/TMZ)***  
***(IDHwt+1p/19q no loss)***

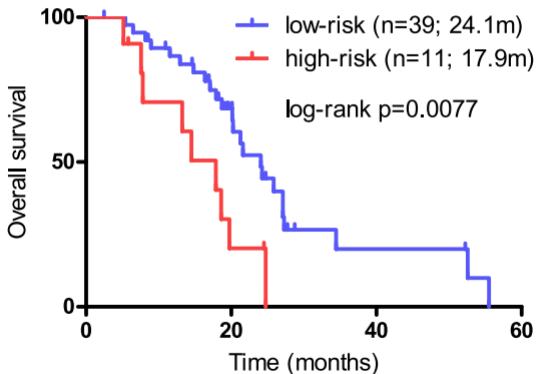

Supplement: Supplementary file 13 — Figure S7. The prognostic performance of the GBM-derived epigenetic signature in a RT/TMZ cohort of GBM-like LGGs without IDH mutations and 1p/19q co-deletion from TCGA; TCGA=The Cancer Genome Atlas; GBM=glioblastoma; RT=radiotherapy; TMZ=temozolomide; LGGs=lower-grade glioma; IDH= isocitrate dehydrogenase. (PDF 227 kb) [file 13148_2019_670_MOESM13_ESM.pdf]
