# Supplementary material for: Novel predictive epigenetic signature for temozolomide in non-G-CIMP glioblastomas
Source: Clin Epigenetics. 2019 May 14;11:76. doi: 10.1186/s13148-019-0670-9 (PMC6515684; doi:10.1186/s13148-019-0670-9)
Supplement: Supplementary file 8 — Figure S4. The predictive performance in terms of PFS outcome; (A) interaction analysis between treatments (with versus without TMZ) and risk subgroups (low-risk versus high-risk) in TCGA-Brennan et al, and (B) interaction analysis between treatments (with versus without TMZ) and risk subgroups (HSPB2 hypomethylation versus hypermethylation) in Xijing cohort; only patients with standard RT regimen were included for analysis in order to reduce potential bias by heterogeneous treatment regimen; SRT= standard radiotherapy; TMZ=temozolomide. (PDF 399 kb) [file 13148_2019_670_MOESM8_ESM.pdf]

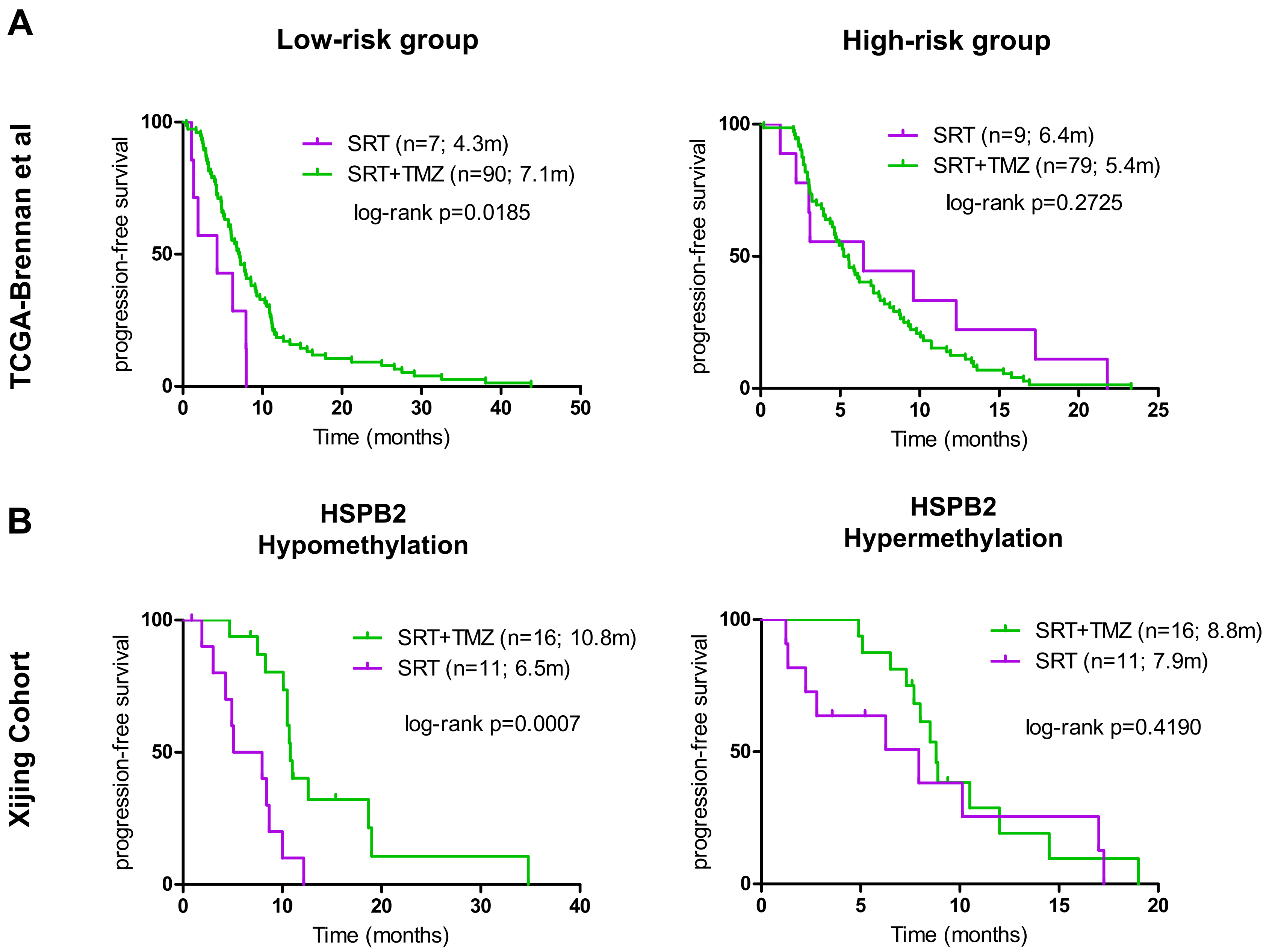
A
B
TCGA-Brennan et al
Xijing Cohort
Low-risk group
SRT (n=7; 4.3m)
SRT+TMZ (n=90; 7.1m)
log-rank p=0.0185
High-risk group
SRT (n=9; 6.4m)
SRT+TMZ (n=79; 5.4m)
log-rank p=0.2725
HSPB2
Hypomethylation
SRT+TMZ (n=16; 10.8m)
SRT (n=11; 6.5m)
log-rank p=0.0007
HSPB2
Hypermethylation
SRT+TMZ (n=16; 8.8m)
SRT (n=11; 7.9m)
log-rank p=0.4190
progression-free survival
Time (months)
