# Supplementary material for: Novel predictive epigenetic signature for temozolomide in non-G-CIMP glioblastomas
Source: Clin Epigenetics. 2019 May 14;11:76. doi: 10.1186/s13148-019-0670-9 (PMC6515684; doi:10.1186/s13148-019-0670-9)

A

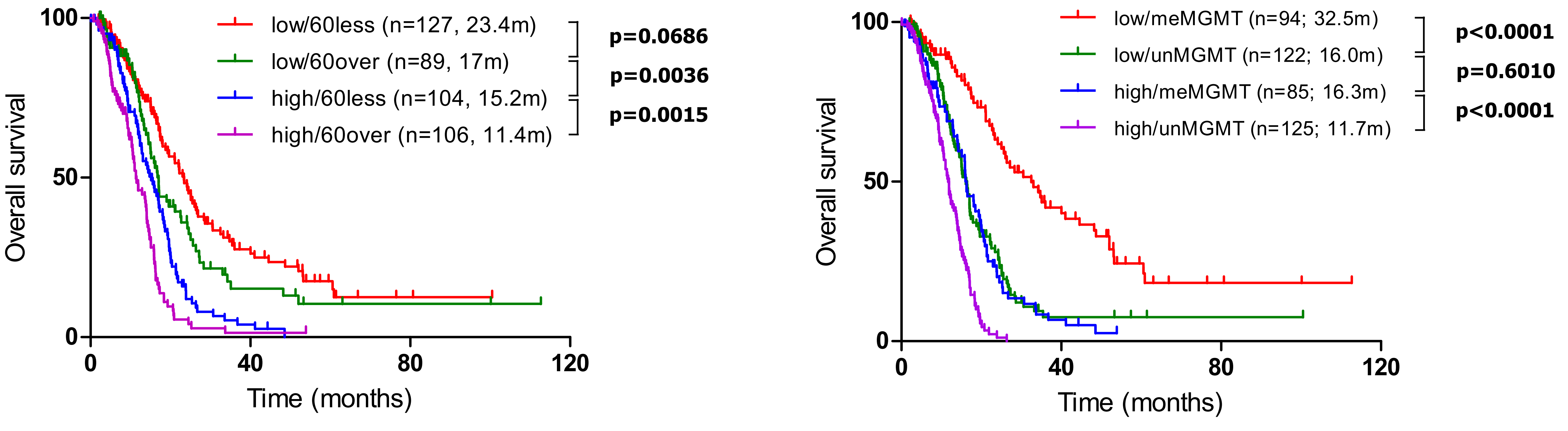

B

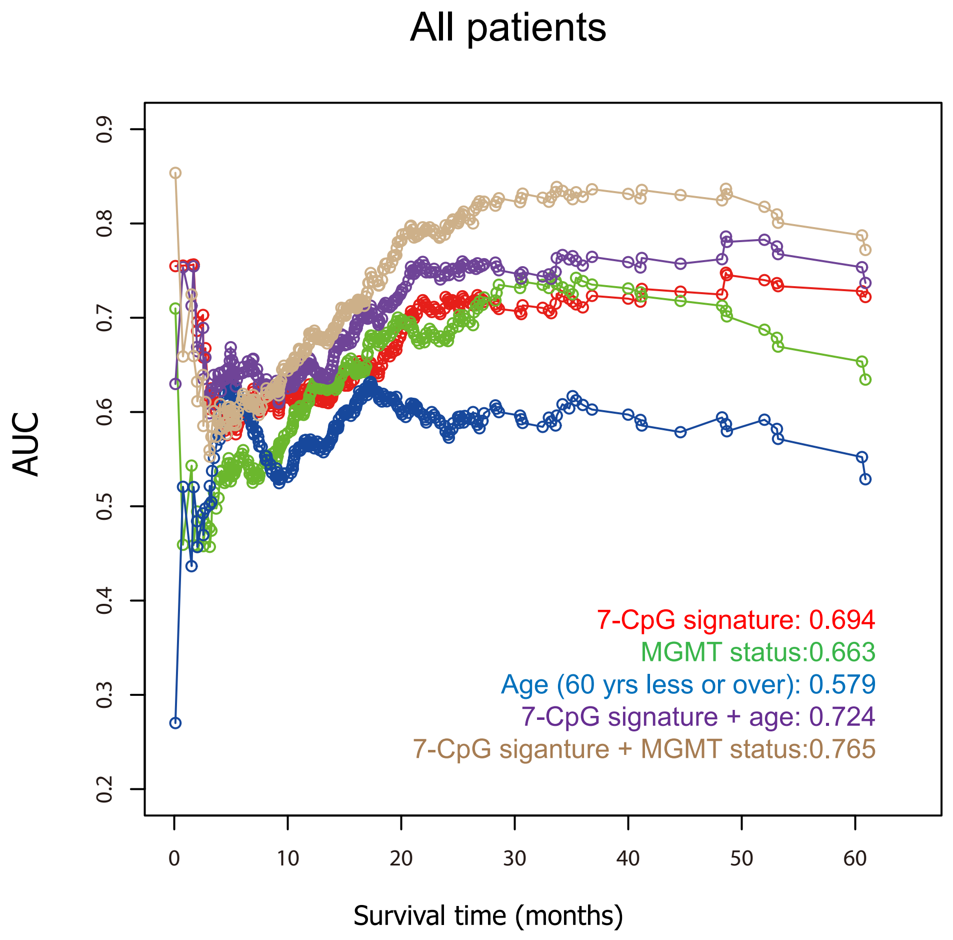

	7-CpG signature	MGMT status	Age (60 yrs less or over)	7-CpG signature + age	7-CpG signature + MGMT status
7-CpG signature	—	—	—	—	—
MGMT status	p<0.0001	—	—	—	
Age (60 yrs less or over)	p<0.0001	p<0.0001	—	—	—
7-CpG signature + age	p<0.0001	p<0.0001	p<0.0001	—	—
7-CpG siganture + MGMT status	p<0.0001	p<0.0001	p<0.0001	p<0.0001	—

C

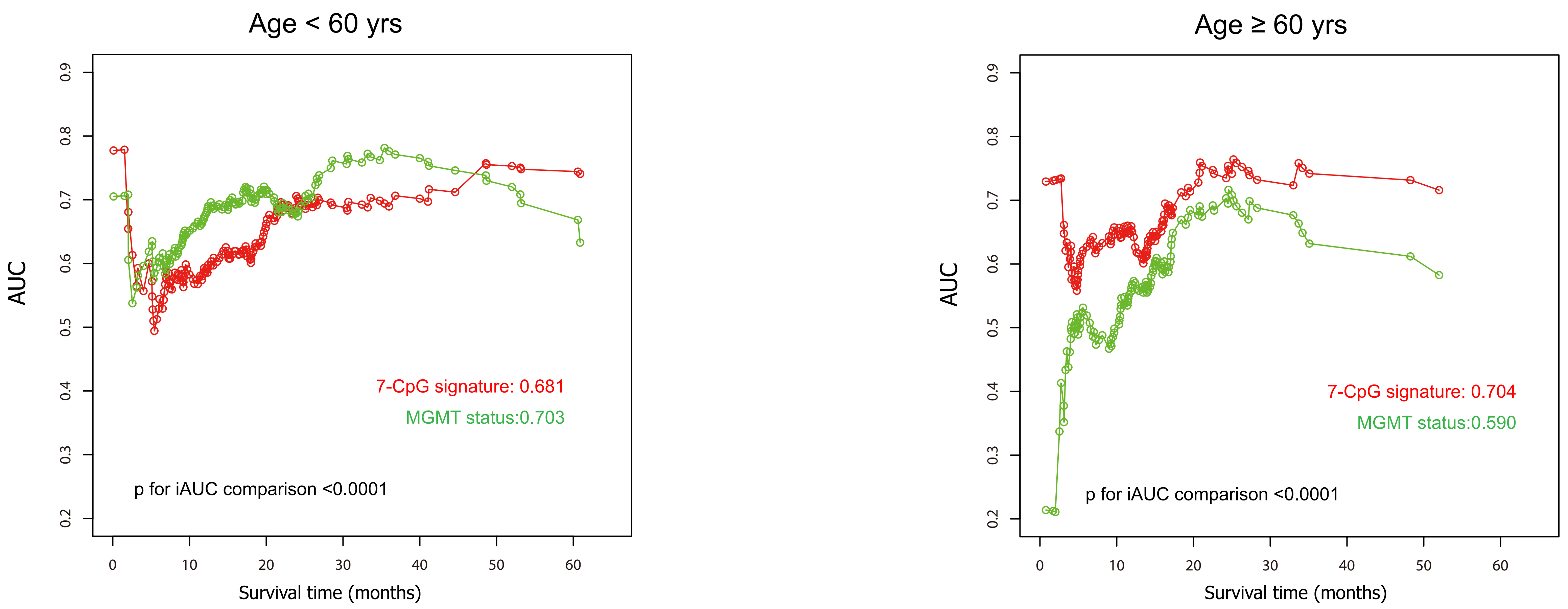

Supplement: Supplementary file 12 — Figure S6. The clinical performance of the RISK-score signature in combination with conventional risk factors in the setting of the combination treatment of RT and TMZ; (A) the risk classification of the RISK-score signature in combination with age (≥60 vs. <60 yrs; left) and MGMT promoter methylation status (right); (B) time-dependent ROC values of the RISK-score signature, age, MGMT promoter methylation status, and their combinations at each time point within all patients who underwent RT/TMZ from RAUH and TCGA-Brennan et al; (C) time-dependent ROC values of the RISK-score signature in comparison with MGMT promoter methylation status in subgroups of different ages at each time point; TCGA=The Cancer Genome Atlas; RAUH=Rennes and Angers University Hospitals; RT=radiotherapy; TMZ=temozolomide; MGMT=O-6-methylguanine-DNA methyltransferase; yrs=years. (PDF 749 kb) [file 13148_2019_670_MOESM12_ESM.pdf]
